# Assessing Opioid Use Disorder Treatments in Trials Subject to Non-Adherence via a Functional Generalized Linear Mixed-Effects Model

**DOI:** 10.3390/ijerph19095456

**Published:** 2022-04-29

**Authors:** Madeleine St. Ville, Andrew W. Bergen, James W. Baurley, Joe D. Bible, Christopher S. McMahan

**Affiliations:** 1 School of Mathematical and Statistical Sciences, Clemson University, Clemson, SC 29634, USA; mstvill@g.clemson.edu (M.S.V.); jdbible@clemson.edu (J.D.B.); 2 Oregon Research Institute, Eugene, OR 97403, USA; abergen@ori.org; 3 BioRealm, LLC, Walnut, CA 91789, USA; baurley@biorealm.ai

**Keywords:** clinical trial, functional general linear mixed model, opiate substitution treatment, opioid urinalysis

## Abstract

The opioid crisis in the United States poses a major threat to public health due to psychiatric and infectious disease comorbidities and death due to opioid use disorder (OUD). OUD is characterized by patterns of opioid misuse leading to persistent heavy use and overdose. The standard of care for treatment of OUD is medication-assisted treatment, in combination with behavioral therapy. Medications for opioid use disorder have been shown to improve OUD outcomes, including reduction and prevention of overdose. However, understanding the effectiveness of such medications has been limited due to non-adherence to assigned dose levels by study patients. To overcome this challenge, herein we develop a model that views dose history as a time-varying covariate. Proceeding in this fashion allows the model to estimate dose effect while accounting for lapses in adherence. The proposed model is used to conduct a secondary analysis of data collected from six efficacy and safety trials of buprenorphine maintenance treatment. This analysis provides further insight into the time-dependent treatment effects of buprenorphine and how different dose adherence patterns relate to risk of opioid use.

## 1. Introduction

There have been nearly 500,000 overdose deaths from opioids in the United States alone in the last 20 years, with associated annual costs exceeding $1 trillion [1]. The treatment of opioid use disorder (OUD) is inherently complex, with clinician assessment of the patient, comorbidities, suitability for one of the three FDA-approved medications, psychosocial counseling and care for comorbidities [2]. One of the two more heavily utilized medications in OUD treatment is buprenorphine, an opioid partial agonist. Studies have been conducted to assess the effectiveness of various formulations and doses of buprenorphine on detoxification, retention in treatment, and on the elimination of illicit opioid use [3]. A meta analysis of clinical trials found that any dose over 2 mg of buprenorphine was useful at retaining patients in treatment but only higher doses (16 mg or more) reduced illicit opioid use [4]. It has been shown that illicit opioid use and the number of weeks abstinent from illicit opioid use are significantly associated with daily buprenorphine adherence [5]. Given the potential interaction between buprenorphine dosing and adherence, further investigations aimed at better understanding these interactions are warranted and will support translational clinical research that seeks to optimize the overall effectiveness of medications for OUD treatment (MOUD).

**Figure 1:**
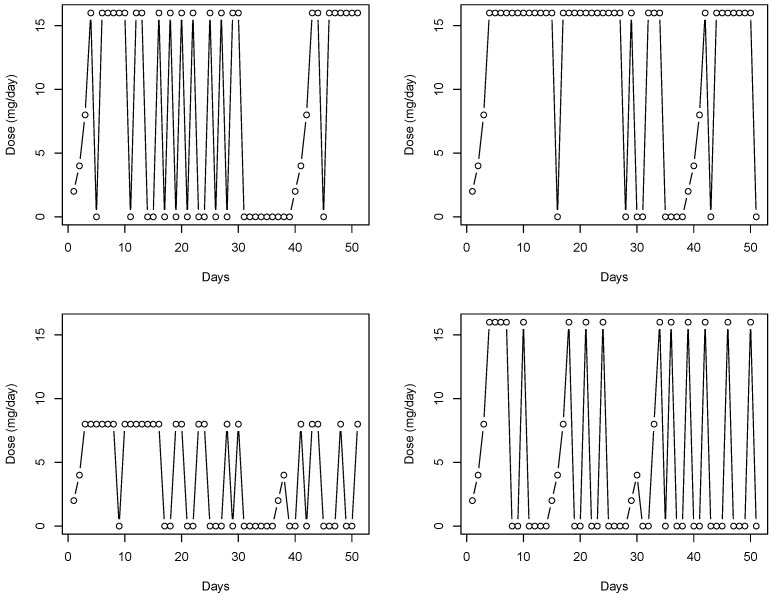
The four figures depict a time series of daily dose of buprenorphine taken by four randomly selected subjects, each coming from the CSP-999 trial, for the first 50 days of the study.

A challenge to being able to accurately assess the effectiveness of MOUD in clinical trials is non-adherence. For example, a multicenter, randomized clinical trial (CSP-999) considered the effectiveness and safety of four buprenorphine dose levels (1, 4, 8, 16, or 32 mg/day), which were administered daily in clinic. Due to the mode of delivery, adherence for this study was directly observed, i.e., patients were either present or absent from the clinic visit. Figure 1 provides a depiction of dose history over a 50 day period for four randomly selected CSP-999 trial patients. In particular, three of the selected patients were assigned to a 16 mg/day dose while the remaining patient was assigned to a 8 mg/day dose. Days when patients missed a dose (i.e., were non-adherent) are represented by a dose level of 0 mg/day. Induction and re-induction after a lapse in dosing can be seen by increasing dose levels from 0 mg/day to the assigned levels. Failing to account for the patterns in adherence depicted in Figure 1 when trying to relate assigned dose to opioid use will obscure the true effect of buprenorphine on illicit opioid use. In particular, not accounting for these patterns will lead to underestimation of the effect (or log-odds) of dose on reducing illicit opioid use.

Given the challenge of non-adherence in substance abuse trials aimed at assessing efficacy of various treatments and dosing protocols, the goals of this paper are two-fold. First, we seek to develop and demonstrate a methodology that can be used to directly acknowledge and account for the effects of adherence when assessing treatment effects. Second, we seek to use our proposed model to assess the effectiveness of buprenorphine as a MOUD. To this end, we compiled, aligned, and harmonized publicly available data from six efficacy and safety clinical trials of buprenorphine maintenance treatment with detailed logs of patient buprenorphine dose. For more on the combined data set and merging steps see [6]. As a part of these trials, patients were expected to attend weekly follow-up clinic visits with opiate urinalysis testing. Dose administration varied across the six trials, with CSP-999 being the only trial requiring daily doses to be self-administered in clinic. Weekly dose adherence for the remaining trials was reconstructed using other available information, e.g., self reported non-adherence and returned pills. Additionally available were a collection of various sociodemographic and substance use variables that were included in the analysis to address potential confounding.

To analyze these data, we develop a generalized linear functional mixed-effects model. The proposed model views daily dose level as a functional covariate whose value reflects the mg/day dose taken, with a value of 0 corresponding to days when the subject is non-adherent. By construction, our model has several key features. First, we can extract an estimate of, and conduct inference about, the dose effect for individuals with strict adherence (i.e., 100% compliance with the prescribed dosing protocol). Second, we can assess the effect of different types of dose self-administration or medication adherence patterns on illicit opioid use. Our model makes use of random effects to account for across trial heterogeneity, and across subject heterogeneity within studies. To complete model fitting, we cast our model into the Bayesian paradigm and develop a custom Markov chain Monte Carlo (MCMC) posterior sampling algorithm.

## 2. Materials and Methods

### 2.1. Generalized Linear Functional Mixed-Effects Model

In what follows, we outline the salient features of our proposed model, which was designed to relate illicit opioid use to time-varying dose adherence while controlling for various demographic and drug use history factors purported to be related to the same. To this end, let $Y_{ij}$, for $j=1,\ldots ,n_{i}$, and i=1,…,m, be a binary indicator such that $Y_{ij}=1$ denotes the event that the ith individual has a positive urinalysis test during the jth clinic visit with urinalysis and $Y_{ij}=0$ otherwise. To relate the observed test data to the available covariates, we posit the following generalized linear functional mixed-effects model

(1) $$\begin{array}{cc} \nu_{ij}=g^{-1}\left(\pi_{ij}\right)=\log\left(\frac{\pi_{ij}}{1-\pi_{ij}}\right) & =\int_{A_{ij}}D_{ij}\left(s\right)\beta \left(s\right)ds+x_{ij}^{T}\alpha +\gamma_{0i}+\gamma_{1k(i)}, \end{array}$$

where g(·) is the logit link function which is used to relate the linear predictor, $\nu_{ij}$, to the probability of relapse, $\pi_{ij}=P\left(Y_{ij}=1\right)$. To elucidate the key feature of our model adopted to capture the effect of dose adherence, we note that the first term on the right-hand side of (1) is the functional component which consists of the time-varying buprenorphine dose curve ($D_{ij}\left(\cdot \right)$, e.g., see Figure 1), the functional coefficient (β(·)), and the time frame ($A_{ij}$) leading up to the urinalysis clinic visit over which the dose levels are allowed to impact the probability of relapse. The remaining components of the model consist of $x_{ij}$ a P-dimensional vector of demographic and drug use history risk factors whose first entry is a one to allow for the usual intercept, α the corresponding vector of regression coefficients, $\gamma_{0i}$ a subject-specific random effect entered into the model to account for the heterogeneity across subjects, $\gamma_{1k(i)}=\gamma_{1k}$ if the *i*th subject is part of the *k*th trial, and $\gamma_{1k}$ is a random effect specified to account for the heterogeneity across trials, k=1,…,K. Herein, the random-effects distributions were taken to be

(2) $$\begin{array}{cc} \gamma_{0i} & \overset{\mathrm{iid}}{∼}N\left(0,\sigma_{0}^{2}\right)\\ \gamma_{1k} & \overset{\mathrm{iid}}{∼}N\left(0,\sigma_{1}^{2}\right), \end{array}$$

and note, here the random effects are taken to be independent given the nesting of subjects within trials. A few comments on the form of the model are warranted. First, through adopting the functional regression framework, we are able to directly acknowledge and estimate the effect of time-varying dose adherence, whereas more traditional variable aggregation techniques (e.g., average dose) fail to acknowledge key trends in dose adherence, e.g., waning adherence from the point of care or weekly patterning. Second, the time window (i.e., $A_{ij}$) should be selected so that the upper bound is just before the *j*th clinic visit with urinalysis for the *i*th individual and that the length of the interval reflects the approximate elimination time for buprenorphine, i.e., buprenorphine doses taken prior to the lower bound are no longer present in the patient’s system and therefore cannot impact the probability of opioid use. Generally speaking, it typically takes five half-lives for a drug to completely leave a subject’s system. Thus, given that the elimination half-life of buprenorphine is 24 to 42 h, we specified a time window consisting of 15 days to more than adequately capture the relevant dose history. Lastly, given the form of the proposed model, we can extract dose effect for individuals with strict adherence to their prescribed dosing regime. To see this, we note that if a subject adheres to the dosing regime, then $D_{ij}\left(s\right)=D_{ij}$ for all *s*. Thus, we would have

$$\begin{array}{c} \begin{array}{cc} \int_{A_{ij}}D_{ij}\left(s\right)\beta \left(s\right)ds=\int_{A_{ij}}D_{ij}\beta \left(s\right)ds & =D_{ij}\int_{A_{ij}}\beta \left(s\right)ds\\ & =D_{ij}\beta^{*} \end{array} \end{array}$$

where $\beta^{*}=\int_{A_{ij}}\beta \left(s\right)ds$. Note, $\beta^{*}$ represent the usual increase in log odds associated with a one unit increase in buprenorphine dose level. Thus, by estimating β(·), we can also estimate $\beta^{*}$.

Estimating the buprenorphine dose effect β(·) in model (1) is challenging from both a theoretical and computational perspective because of its infinite dimension. To reduce the number of unknown parameters needed to be estimated while also maintaining adequate modeling flexibility, we approximate β(·) using B-splines [7]. This leads to the following representation of β(·):

(3) $$\beta (\cdot )=\sum_{\ell =1}^{L}\eta_{\ell }b_{\ell }\left(\cdot \right),$$

where $b_{\ell }\left(\cdot \right)$ is a spline basis function and $\eta_{\ell }$ is the corresponding spline coefficient, for ℓ=1,…,L. The *L* spline basis functions are fully determined once the degree and knot set are specified, thus the only unknown parameters in (3) are the spline coefficients. In specifying the basis functions, the degree controls the overall smoothness of the basis functions and the number of knots determines the overall modeling flexibility; for further discussion see [7]. We suggest selecting a relatively large knot set (e.g., 5–6 knots) and then regularizing the estimation of the spline coefficients through the methodology outlined below.

Using the spline representation of β(·) depicted in (3), we can re-express the functional component in model (1) as follows

(4) $$\begin{array}{cc} \int_{A_{ij}}D_{ij}\left(s\right)\beta \left(s\right)ds & =\int_{A_{ij}}D_{ij}\left(s\right)\left(\sum_{\ell =1}^{L}\eta_{\ell }b_{\ell }\left(s\right)\right)ds\\ \; & =\sum_{\ell =1}^{L}\left(\int_{A_{ij}}D_{ij}\left(s\right)b_{\ell }\left(s\right)ds\right)\eta_{\ell }\\ \; & :=m_{ij}^{T}\eta , \end{array}$$

where $m_{ij}$ is an *L*-dimensional vector whose *ℓ*th element is $m_{ij\ell }=\int_{A_{ij}}D_{ij}\left(s\right)b_{\ell }\left(s\right)ds$ and $\eta ={\left(\eta_{1},\ldots ,\eta_{L}\right)}^{\prime }$. Thus, the linear predictor of our model can be expressed as

(5) $$\begin{array}{c} \nu_{ij}=m_{ij}^{T}\eta +x_{ij}^{T}\alpha +\gamma_{0i}+\gamma_{1k(i)}. \end{array}$$

### 2.2. Prior Specification

To facilitate both parameter estimation and inference, we cast our problem into the Bayesian paradigm. The first step in this process involves specifying prior distributions for all unknown parameters. Given the complexities of our problem, priors are chosen to regularize the estimation of the parameters. In particular, the prior for the spline coefficients is designed to encourage smoothness in the functional estimate while the prior for the regression coefficients is meant to “shrink” unimportant variables toward zero. In what follows, we briefly expand on these specifications.

To avoid overfitting issues and to encourage smooth functional estimates, herein we adopt a prior for the spline coefficients which leverages a covariance structure inspired by the usual roughness penalty [8]. This common penalty encourages smoothness by penalizing for abrupt changes in the function through the following:

$$\begin{array}{c} \int {\left[\beta^{\left(2\right)}\left(s\right)\right]}^{2}ds=\eta^{T}R\eta , \end{array}$$

where $\beta^{\left(2\right)}\left(\cdot \right)$ denotes the second derivative of β(·) and R is an L×L matrix with entries $R_{\ell \ell^{\prime }}=\int b_{\ell }^{\left(2\right)}\left(s\right)b_{\ell^{\prime }}^{\left(2\right)}\left(s\right)ds$ with $b_{\ell }^{\left(2\right)}\left(\cdot \right)$ being the second derivative of $b_{\ell }\left(\cdot \right)$. Note, the spline representation adopted for β(·) is key to being able to represent this penalty as the quadratic form depicted above; for details of this derivation see [8]. Capitalizing on the structure of this penalty and the duality that exists between regularized estimation and prior distributions in the Bayesian paradigm, we specify the following smoothing penalty inspired prior distribution for η:

$$\begin{array}{cc} \eta & ∼N(0,\lambda R^{-1})\\ \lambda & ∼Inv-Gamma(a_{\lambda },b_{\lambda }). \end{array}$$

In the prior specification above, the additional variance parameter λ governs the amount of smoothness and controls the trade off between over and underfitting the data.

To aid in variable selection, we adopt the generalized double Pareto shrinkage prior, proposed by [9], for all of the regression coefficients with the exception of the intercept, i.e., we specify

$$\begin{array}{cc} \alpha_{0} & ∼N(0,\tau_{0})\\ \alpha_{p} & ∼GDP\left(\psi =b_{\delta }/a_{\delta },a_{\delta }\right),\;\mathrm{for}\;p=1,\ldots ,P-1, \end{array}$$

where $\mathrm{GDP}(\psi ,a_{\delta })$ refers to the generalized double Pareto distribution [9]. Under these prior choices, setting $\tau_{0}$ to be large provides a vague prior on $\alpha_{0}$, while the hyperparameters $a_{\delta }>0$ and $b_{\delta }>0$ govern the amount of shrinkage. In particular, these parameters control the dispersion, with $a_{\delta }$ controlling the heaviness of the tails of the distribution. A typical default specification, and the one adopted herein, is to set $a_{\delta }=b_{\delta }=1$ which leads to Cauchy-like tail behavior which is known to have desirable Bayesian robustness properties [9].

Finally, we place inverse gamma priors on the variance components of the random effects, i.e., we specify

$$\begin{array}{cc} \sigma_{q}^{2} & ∼Inv-Gamma(a_{q},b_{q}),\;q=0,1. \end{array}$$

This specification is common among the literature and it leads to a proper posterior [10]. Based on the prior specifications outlined above, we develop a Markov chain Monte Carlo (MCMC) sampling algorithm which facilitates both posterior estimation and inference. In what follows, we provide a brief overview of this algorithm and its construction.

### 2.3. Data Augmentation and Posterior Sampling

With ease of implementation and computational efficiency in mind, herein we outline the construction of a posterior sampling algorithm that consists solely of Gibbs steps [11]. To accomplish this, we consider a two-stage data augmentation process. The first stage follows the work of [12], and introduces carefully constructed Pólya-Gamma latent random variables so that the logistic function can be hierarchically expressed as a scale mixture of normals, where the mixing distribution is Pólya-Gamma; for further details, see [12]. The second stage decomposes the generalized double Pareto shrinkage prior as a scale mixture of normals; for further discussion see [9]. For the specific details of this two-stage data augmentation process, see Appendix A.

The data augmentation scheme outlined above leads to the following full conditionals

$$\begin{array}{cc} \alpha |Y,w,\eta ,\gamma_{0},\gamma_{1},\tau & ∼N(\mu_{\alpha },\Sigma_{\alpha })\\ \eta |Y,w,\alpha ,\gamma_{0},\gamma_{1},\lambda & ∼N(\mu_{\eta },\Sigma_{\eta })\\ \lambda |\eta & ∼Inv-Gamma(a_{\lambda }^{*},b_{\lambda }^{*})\\ \sigma_{q}^{2}|\gamma_{q} & ∼Inv-Gamma(a_{q}^{*},b_{q}^{*})\\ w_{ij}|\alpha ,\eta ,\gamma_{0i},\gamma_{1k(i)} & ∼PG(b_{\delta }^{*}/a_{\delta }^{*},a_{\delta }^{*})\\ \tau_{p}|\alpha_{p},\delta_{p} & ∼Inv-Gaussian(a_{\tau_{p}}^{*},b_{\tau_{p}}^{*})\\ \delta_{p}|\alpha_{p} & ∼Gamma(a_{\delta_{p}}^{*},b_{\delta_{p}}^{*}), \end{array}$$

where the specific form of the parameters of these distributions are given in Appendix A. These full conditionals were used to construct an MCMC algorithm in the usual manner; for further discussion see [11].

## 3. Secondary Data Analysis of Buprenorphine Efficacy

### 3.1. Trial Data

Clinical trial data for this analysis were sourced from the Clinical Trials Network (CTN) at NIDA’s Data Share resource (https://datashare.nida.nih.gov/ (accessed on 5 September 2017)). Using the search keyword *opiate*, we identified 10 efficacy and safety trials involving detoxification or maintenance treatment of DSM-IV opioid dependence. We selected six efficacy and safety trials focused on buprenorphine maintenance treatment for analysis. Detailed information on these trials are provided in Table A1.

### 3.2. Patient Characteristics

The data consist of 55,739 urinalysis results from 3022 subjects who participated in one of the six aforementioned clinical trials aimed at assessing the efficacy of buprenorphine for treating OUD. The number of urinalyses (i.e., urine drug screens for opioids) per subject ranged from 1 to 60 urinalyses, while the mean number of urinalyses per subject was 18.44 and the median was 18. The data were harmonized across the six trials and candidate predictors with a missingness greater than 25% were filtered out. This resulted in 18 demographic, sociodemographic, and substance use variables (excluding prescribed buprenorphine dose, handled by the functional component of the model). Missing demographic, sociodemographic, and substance use variables were imputed using the regularized iterative factorial analysis for mixed data (qualitative and quantitative variables) algorithm [13], implemented by the imputeFAMD function in the missMDA R package. Summaries of the retained variables (with imputed values included) are given in Table 1. The daily dose of buprenorphine taken by each patient was either reported (CSP-999) or inferred from alternate information. Daily dose could vary throughout time for a variety of reasons, e.g., adherence, induction, re-induction after lapse in dosing, and modification by a provider’s clinical judgement.

Given the number of demographic and substance use variables considered, the reference group is specifically white men with a high school diploma who are employed full time doing skilled manual labor, married and living with a partner or child, and their primary mode of opioid use being intravenous, with a history of heroin, cocaine, alcohol and marijuana use and no history of methamphetamine, tranquilizer, or PCP use. The mean age, income, and years of opioid use are 36.14 years, $20,834 per year and 8.23 years, respectively (presented in Table 1), while the mean dose is 12.65 mg/day (presented in Table 2). When we discuss conditional probabilities of relapse, comparisons will be made with respect to this hypothetical individual in the reference group by changing specific variables as noted.

### 3.3. Functional General Linear Mixed Model

The outcome variable in this analysis was the urinalysis test result for illicit opioid use (1 = positive drug screen vs. 0 = negative drug screen). Through the model in (1), we relate the daily dose patterns leading up to the clinic visit with urinalysis, while controlling for the 18 demographic, sociodemographic, and substance use variables detailed in Table 1. For the functional dose component in model (1), the time trajectory was chosen to be the 15 days leading up to the current urinalysis clinic visit and, for the B-spline basis expansion of the coefficient function in (3), we specify the degree to be 3 to construct cubic basis functions. Two interior knots were placed at the 33.33th and 66.67th percentiles of our 15 day time range. This leads to five fully determined spline basis functions and, hence, five spline basis coefficients to estimate. For the priors outlined in Section 2.2, we take $\tau_{0}=1000$ to specify a vague prior on the global intercept $\alpha_{0}$ and let $a_{0}=b_{0}=0.001$, $a_{1}=b_{1}=0.005$, $a_{\delta }=1,b_{\delta }=1$, $a_{\lambda }=1,b_{\lambda }=0.005$. These hyperparameter values are chosen so to produce uninformative, proper prior specifications. For sampling, we retain 5000 MCMC iterates after a burn-in of 5000 samples. Convergence of the MCMC chains was assessed in the usual manner, i.e., trace plots. To summarize our analysis, we report the estimated posterior means (point estimates of the effects), estimated posterior standard deviations (measures of uncertainty), and 95% equal-tailed credible intervals.

**Figure 2:**
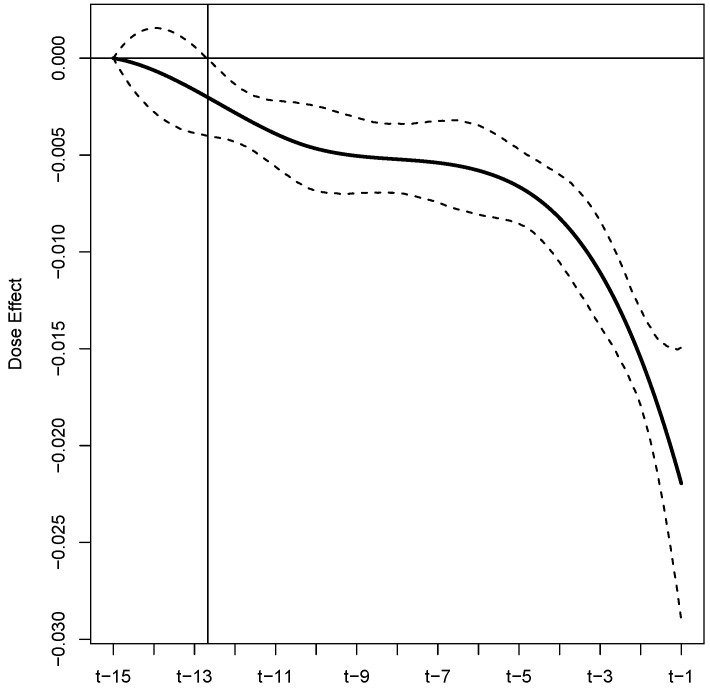
Estimated buprenorphine dose effect for the 15 days leading up to a urinalysis test, with 95% equal-tailed credible interval limits displayed as black dashed lines. The intersection of the vertical and horizontal lines is the point at which the credible interval is entirely below zero, marking the point where the dose effect becomes significant.

Figure 2 summarizes the estimated functional coefficient $\hat{\beta }\left(t\right)$ (black solid line), which represents the buprenorphine daily dose effect for the 15 days leading up to a clinic visit with urinalysis. The dashed lines are the 95% credible interval limits. On the horizontal axis, if *t* is the day of the current urinalysis clinic visit, then t−15 represents 15 days prior and t−1 represents one day prior. Table 3 reports the demographic and substance use variables that were found to be significant. Of the 54 fixed effects, four were deemed to be important by the model (i.e., their estimated credible intervals did not contain zero). Table 3 summarizes these significant factors by reporting the estimated posterior means (point estimate of the effect), estimated posterior standard deviations (measure of uncertainty), and 95% equal-tailed credible intervals. The analogous results for the full set of demographic and substance use variables are provided in Table A2 and Table A3 in Appendix C.2.

As previously stated, daily dose adherence was only directly recorded for patients in the CSP-999 trial. Specifically, while the assigned daily dose of buprenorphine was recorded as a part of the five other trials, adherence was not. For these trials, dose adherence was reconstructed using other available information, e.g., self reported non-adherence and returned pills. To examine how the buprenorphine dose reconstruction could have impacted our results, we reran our analysis on data from the CSP-999 trial only. A summary of these results can be found in Appendix C.

To extract an estimate of dose effect for subjects that were strictly adherent to their assigned dosing regime, we compute the following integral for each realization β(s), denoted $\beta^{\left(g\right)}\left(s\right)$, drawn from the posterior

$$\begin{array}{c} \beta^{*(g)}=\int_{A_{ij}}\beta^{\left(g\right)}\left(s\right)ds, \end{array}$$

with $\beta^{*(g)}$ being a posterior realization of $\beta^{*}$. Table 4 provides a summary of these results for both the full and reduced (CSP-999) analysis to include the posterior mean estimate (point estimate of the effect), estimated standard deviation of the posterior (measure of uncertainty), and 95% equal-tailed credible interval.

## 4. Discussion

The primary focus of our analysis is two-fold. First, we wish to demonstrate a novel approach to account for non-adherence that commonly arises in medication-assisted treatment trials; especially those targeting substance use disorders. Second we wish to refine the understanding of the effectiveness of buprenorphine as a MOUD, while accounting for the potential non-adherence of study patients. To accomplish both of these tasks, we investigated the influence of multiple demographic, sociodemographic, drug use history, and treatment variables on the risk of illicit opioid use with publicly available individual patient data from six Federally sponsored buprenorphine efficacy and safety trials. To acknowledge and account for patterns of non-adherence, we conceptualized the daily dose histories of the study patients as a functional covariate and we estimated an associated functional effect. A summary of this estimated functional is provided in Figure 2. From these results, we identify several key findings. First, these results suggest that buprenorphine, as an MOUD, significantly reduces the risk of illicit opioid use. This can be seen from the fact that the point estimates, and associated credible intervals, are all below zero, i.e., the integral over the the product of this functional and $D_{ij}\left(\cdot \right)\geq 0$ results in a negative quantity. Second, we find that dose history extending to approximately 12.5 days prior to an opioid screening visit is significantly related to the risk of short-term lapses. Third, the risk of illicit opioid use is related to dosing adherence patterns throughout the considered 15 day window leading up to the urinalysis, although, recent patterns have more influence. This can be seen from the decreasing nature of the functional estimate, especially for the five (approximately) days before the urinalysis test. Fourth, based on our estimated functional, we are able to extract an estimate of dose effect for subjects that were strictly adherent to their assigned dosing protocol. Based on this approach, we estimate that the log-odds of short-term lapse decreases by 0.09 with every 1 mg/day increase in dose; see Table 4. This new assessment of the efficacy of buprenorphine as an OUD treatment is unobscured by the effects of non-adherence and leverages six NIDA-sponsored efficacy and safety trials to render its conclusions.

When examining the association between risk of illicit opioid use and the other demographic, sociodemographic, and drug use history variables, four of the 54 were found to be significant. In particular, we find that increasing age is protective, while being unemployed, a drug use history of using heroine, and a drug use history of using opioids intravenously are associated with an increased risk of illicit opioid use. A similar finding that increasing age is associated with no positive urine drug screen was recently reported in an analysis of Veterans Administration patients undergoing buprenorphine treatment [14]. The protective nature of employment for patients in recovery [15] is concordant with unemployment being identified as a risk factor for illicit opioid use. Older age, no heroine use history and no IV drug use have already been reported as protective with respect to successful opioid use outcomes (abstinent during week 24 and ≥2 of the previous 3 weeks) in a secondary data analysis of the Prescription Opioid Addiction Treatment Study (POATS or CTN-0300) [16,17], one of six CTN trials included in this study. Notably, the largest protective effect we observed was “primary mode of opioid abuse” with the log-odds of short-term lapse decreasing by 1.32 when the primary mode of abuse is oral. This could be attributable to the severity of the opioid use disorder, with intravenous use being a hallmark of more severe cases.

When examining the results of the sensitivity analysis (see Appendix C) of the CSP-999 trial only, we note several similarities and differences. Importantly, the full and CSP-999 analysis came to virtually the same conclusions with regard to the efficacy of buprenorphine as an OUD treatment. In particular, the estimates of β(·) are not statistically different from each other. However, the estimate from the full analysis is slightly attenuated toward zero when compared to the CSP-999 only analysis. This feature can also be observed in the effect estimate reported in Table 4. A plausible explanation for this would be that our approach to reconstructing dose histories for the study patients, though effective, was not perfect, and therefore introduced “measurement error” into this variable. A hallmark of measurement error is the attenuation of effect estimates toward zero, e.g., see [18]. When comparing the estimated effects of demographic, sociodemographic, and substance use variables we find that most are not statistically different, yet there are differences in those deemed to be significant by the two analyses. These differences are likely attributable to increased precision due to larger sample sizes in the full analysis and differences in the demographic distribution across the full and reduced data.

We also acknowledge the lack of other risk factors that could be used to better understand/predict short-term lapse. Inclusion of time-varying predictors such as current stress levels, occurrences of major life events (e.g., familial death and loss of job), and other psychological measures would undoubtedly enhance our model. However, the impact of not having these variables is mitigated by the adoption of subject specific random effects.

Importantly, this study was specifically aimed at estimating the effectiveness of recent buprenorphine treatment at reducing short-term lapse. With that being said, this analysis did not consider adherence and its impact on illicit opioid use over longer periods of time and the subsequent associations with OUD-related adverse outcomes, which is a far more complex problem. Studies aimed at these more long-term outcomes could reveal OUD treatment strategies that would be poised to positively impact public health. That is, there have been nearly 500,000 overdose deaths from opioids in the United States alone in the last 20 years [1]. Further, OUD-related mortality appears to be increasing. Specifically, the CDC estimates that overdose deaths from opioids increased to 75,673 in the 12-month period ending in April 2021, up from 56,064 in 2020 [19]. Moreover, less than one-third of patients enrolled in comprehensive health care with current OUD are being treated with one of three approved medications for OUD [20]. Extended MOUD treatment (>1 vs. ≤1 year) appears to reduce mortality [21]. Thus, more in-depth studies relating MOUD treatment to long-term outcomes has the potential to identify OUD treatment strategies that can be more effectively utilized to treat this epidemic and shift current clinical practice. Future research efforts will be aimed at studying these more complex topics related to dose, adherence and treatment outcomes and their association with OUD-related mortality rates.

## 5. Conclusions

Inspired by adherence issues in six efficacy and safety clinical trials of buprenorphine maintenance treatment, this work proposed a generalized linear functional mixed-effects model that can acknowledge and account for the effects of adherence when assessing treatment effect. The proposed model was applied to the six motivating clinical trials in an effort to better refine understanding about the time-dependent effect that buprenorphine has on treating OUD. In particular, we find that dose history approximately 12.5 days prior to an opioid screening visit is significantly related to the risk of short-term lapses, with the more recent history being more impactful. Further, we are able to extract an estimate of dose effect that is not obscured by adherence issues. That is, we estimate that the log-odds of short-term lapse decreases by 0.09 with every 1 mg/day increase in buprenorphine dose.

## Figures and Tables

**Table 1:** Sociodemographic characteristics and drug use history for the individuals used in the analysis.

*Demographics *			*Sociodemographics*		
**Age **	Mean	SD	**Income**	Mean	SD
	36.14	9.85		20,834	30,025
**Gender**	N	%	**Employment History**	N	%
Male	2017	67	Skilled Manual	889	29
Female	1005	33	Never Gainfully	653	22
**Race**	N	%	Machine Operator	445	15
White	2001	66	Clerical/Sales	407	13
Hispanic	495	16	Administrative	239	8
Black	422	14	Unskilled	235	8
American Indian	50	2	Business Manager	101	3
Asian	48	2	Executive	53	2
Other	6	<1	**Work Type**	N	%
			Full time	1758	58
***Drug Use History***			Unemployed	582	19
**Years of Opiate Abuse**	Mean	SD	Irregular PT	284	9
	8.23	8.41	Regular PT	232	8
**Heroin Use**	N	%	Retired	84	3
YES	2354	78	Student	64	2
NO	668	22	Controlled	17	<1
**Mode of Opiate Abuse**	N	%	Military	1	<1
IV	1710	57	**Education**	N	%
Snort	1089	36	High School	1456	48
Oral	122	4	Partial College	829	27
Smoking	74	2	Partial High School	304	10
Other	22	1	Standard College	213	7
Sublingual	5	<1	Junior High School	116	4
**Cocaine Use**	N	%	Complete Graduate School	89	3
YES	1837	61	Less than 7th Grade	15	1
NO	1185	39	**Marital Status**	N	%
**Meth Use**	N	%	Married	1038	34
NO	2304	76	Never Married	1014	33
YES	718	24	Divorced	602	20
**Alcohol Use**	N	%	Separated	261	9
YES	1891	63	Widowed	87	3
NO	1131	37	Remarried	20	1
**Tranquilizer Use**	N	%	**Living Arr**	N	%
NO	1997	66	Partner and Child	1251	41
YES	1025	34	Partner Only	537	18
**Marijuana Use**	N	%	Parents	294	10
YES	1953	65	Family	263	9
NO	1069	35	Friends	255	8
**PCP Use**	N	%	Alone	190	6
NO	2545	84	Child Only	183	6
YES	477	16	Controlled	25	1
			No Stable	24	1

**Table 2:** Treatment and outcome characteristics of individuals used in the analysis. Urinalysis is a binary indicator that takes value 1 to denote a positive opioid drug screen and 0 otherwise.

	Mean	Median	Range
Daily Dose	12.65	14	0–90
Days in Trial	112.70	87	1–527
Urinalysis	0.41	0	0–1

**Table 3:** Analysis results: Summary includes the posterior mean estimates (Est), the estimated posterior standard deviations (ESE), and the estimated 95% equal-tailed credible intervals (CI95) for the significant fixed effects.

Variable	Est	ESE	CI95
Intercept	2.05	0.27	(1.54, 2.61)
Age	−0.02	0.01	(−0.03, −0.01)
**Work Type** (Ref: Full time)			
Unemployed	0.33	0.14	(0.05, 0.59)
**Heroine Use** (Ref: YES)			
No Heroine Use	−0.57	0.21	(−1.00, −0.16)
**Mode of Opioid Abuse** (Ref: IV)			
Oral	−1.32	0.23	(−1.78, −0.88)

**Table 4:** Analysis results: Summary includes the posterior mean estimate (Est), the estimated standard deviation of the posterior (ESE), and the estimated 95% equal-tailed credible interval (CI95) for the dose effect (i.e., $\beta^{*}$) for the full and reduced (CSP-999) analysis.

	Est	ESE	CI95
All Trials	−0.09	<0.01	(−0.09, −0.08)
CSP-999	−0.11	0.01	(−0.12, −0.10)

**Figure A1:**
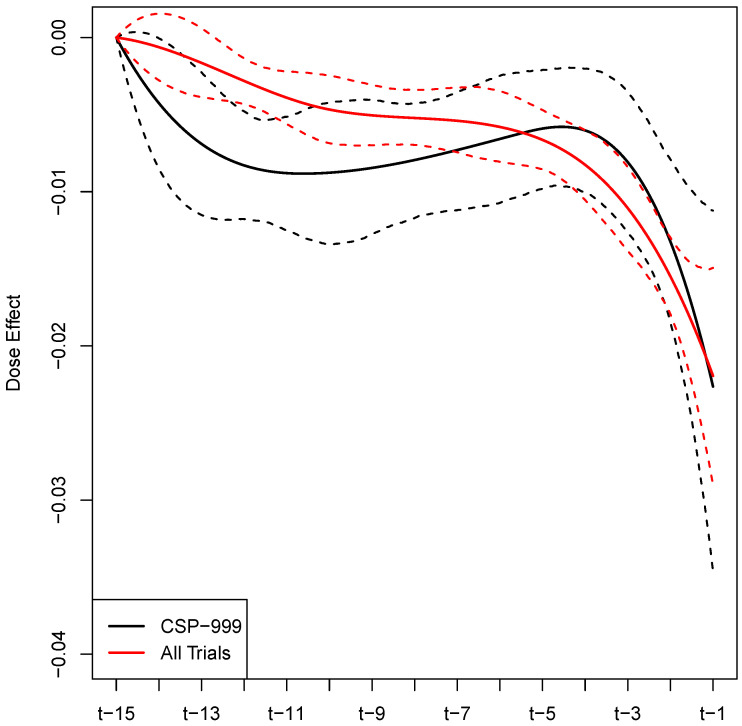
Estimated buprenorphine dose effect for the 15 days leading up to a urinalysis test, with 95% equal-tailed credible interval limits.

## Data Availability

Clinical trial data are available from the Clinical Trials Network (CTN) at NIDA’s Data Share resource (https://datashare.nida.nih.gov/ (accessed on 5 September 2017)). Using the search keyword *opiate*, we identified 10 efficacy and safety trials involving detoxification or maintenance treatment of DSM-IV opioid dependence. We selected six efficacy and safety trials focused on buprenorphine maintenance treatment for analysis (Table A1).

## Appendix A. Posterior Distributions

We assume conditional independence given the covariate effects and random effects and observe that $Y_{ij}$ depends on the model parameters only through the linear predictor, $\nu_{ij}$. Hence, the likelihood can be expressed as

$$p\left(Y|\nu \right)\propto \prod_{i,j}g{\left(\nu_{ij}\right)}^{Y_{ij}}{\left\{1-g\left(\nu_{ij}\right)\right\}}^{1-Y_{ij}},$$

where g(·) is defined to be the logit link function.

We develop a two-stage data augmentation process to construct a posterior sampling algorithm consisting only of Gibbs steps. In the first stage, we exploit a hierarchical representation of the proposed data model by introducing Póyla-Gamma latent random variables $w_{ij}$; for further details, see [12]. Under this specification, the joint density of the observed and latent data for the *i*th individual is given by

$$p\left(Y_{i},w_{i}|\nu_{i}\right)\propto \exp\left\{-\frac{1}{2}{\left(h_{i}-\nu_{i}\right)}^{T}W_{i}\left(h_{i}-\nu_{i}\right)\right\}\times \prod_{j}\xi \left(w_{ij}\right),$$

where $h_{i}={\left(\kappa_{i1}/w_{i1},\ldots ,\kappa_{in_{i}}/w_{in_{i}}\right)}^{T}$ are synthetic responses with $\kappa_{ij}=Y_{ij}-1/2$, $W_{i}=\mathrm{diag}\left(w_{i}\right)$, $\xi \left(w_{ij}\right)=f\left(w_{ij}|1,0\right)\exp\left\{\kappa_{ij}^{2}/\left(2w_{ij}\right)\right\},$ and $f(w_{ij}|a,b)$ denotes the Pólya-Gamma density with parameters (a,b); for further details, see [12].

Attention is now turned to the second stage of the data augmentation process and the construction of the hierarchical representation of the joint posterior distribution. Recall from Section 2.2, we specify a generalized double Pareto shrinkage prior for all of the regression coefficients with the exception of the intercept, i.e.,

$$\begin{array}{cc} \alpha_{0} & ∼N(0,\tau_{0}),\\ \alpha_{p} & ∼GDP\left(\psi =b_{\delta }/a_{\delta },a_{\delta }\right),\;\mathrm{for}\;p=1,\ldots ,P-1. \end{array}$$

As noted by Proposition 1 of [9], the generalized double Pareto shrinkage prior can be represented as a scale mixture of normal distributions. Thus, for the regression coefficients, the following hierarchical representation provides the same prior specifications as those given above:

$$\begin{array}{cc} \alpha & ∼N(0,T),\;\mathrm{where}\;T=\mathrm{diag}\left(\tau \right),\;\tau ={\left(\tau_{0},\tau_{1},\ldots ,\tau_{P-1}\right)}^{T}\\ \tau_{p} & \overset{\mathrm{ind}.}{∼}Exponential\left(\delta_{p}^{2}/2\right),\;\mathrm{for}\;p=1,\ldots ,P-1\\ \delta_{p} & \overset{\mathrm{iid}}{∼}Gamma\left(a_{\delta },b_{\delta }\right),\;\mathrm{for}\;p=1,\ldots ,P-1. \end{array}$$

The $\delta_{p}$s are the global shrinkage parameters, while the $\tau_{p}$s are the local shrinkage parameters and override the impact of the global shrinkage components for the variable fixed effects that are not near zero [9].

In deriving the full conditional distributions, for notational convenience, a dot · is used as shorthand for all the parameters one is conditioning on, e.g., we may write the posterior p(α|Y,w,η,γ,τ) as p(α|·).

We begin by deriving the full conditional distribution for the spline coefficients η, based on the smoothing penalty inspired prior distribution outlined in Section 2.2. Letting $h^{\eta }={\left(h_{1}^{\eta },\ldots ,h_{n}^{\eta }\right)}^{T}$, where $h_{i}^{\eta }:=h_{i}-X_{i}\alpha -1_{n_{i}}\gamma_{0i}-1_{n_{i}}\gamma_{1k(i)},$ and $M={\left(M_{1},\ldots ,M_{n}\right)}^{T}$, W=diag(w),

$$\begin{array}{cc} p(\eta |\cdot ) & \propto p(Y,w|\nu )p(\eta |\tau )\\ \; & \propto \exp\left\{-\frac{1}{2}{\left(h^{\eta }-M\eta \right)}^{T}W\left(h^{\eta }-M\eta \right)\right\}\times \exp\left\{-\frac{1}{2}\eta^{T}\left(\lambda^{-1}R\right)\eta \right\}\\ \; & \propto \exp\left\{-\frac{1}{2}{\left(\eta -\mu_{\eta }\right)}^{T}{\left(\Sigma_{\eta }\right)}^{-1}\left(\eta -\mu_{\eta }\right)\right\}, \end{array}$$

where $\Sigma_{\eta }={\left(M^{T}WM+\lambda^{-1}R\right)}^{-1}$ and $\mu_{\eta }=\Sigma_{\eta }M^{T}Wh^{\eta }$. Recognizing this as the kernel of a normal density, we have

$$\eta |\cdot ∼N(\mu_{\eta },\Sigma_{\eta }).$$

Further, the full conditional distribution for the variance parameter λ associated with the smoothing prior for the spline coefficients η is derived as follows.

$$\begin{array}{cc} p(\lambda |\cdot ) & \propto p(\eta |\lambda )p(\lambda )\\ \; & \propto {\left(\lambda^{-1}\right)}^{L/2}\exp\left\{-\frac{\lambda^{-1}}{2}\eta^{T}R\eta \right\}\times {\left(\lambda^{-1}\right)}^{a_{\lambda }-1}\exp\left\{-\lambda^{-1}b_{\lambda }\right\}\\ \; & \propto {\left(\lambda^{-1}\right)}^{a_{\lambda }^{*}-1}\exp\left\{-\lambda^{-1}b_{\lambda }^{*}\right\}, \end{array}$$

where $a_{\lambda }^{*}=a_{\lambda }+L/2$ and $b_{\lambda }^{*}=b_{\lambda }+0.5\cdot \eta^{T}\eta$. Recognizing this as the kernel of a Gamma density, we find that

$$\lambda |\cdot ∼Inv-Gamma(a_{\lambda }^{*},b_{\lambda }^{*}).$$

Given the hierarchical representation of the priors placed on the regression coefficients, let $h^{\alpha }={\left(h_{1}^{\alpha },\ldots ,h_{n}^{\alpha }\right)}^{T}$ where $h_{i}^{\alpha }:=h_{i}-M_{i}\eta -1_{n_{i}}\gamma_{0i}-1_{n_{i}}\gamma_{1k(i)}$, and let $X={\left(X_{1},\ldots ,X_{n}\right)}^{T}$. We derive the full conditional distribution for the regression coefficients α as follows.

$$\begin{array}{cc} p(\alpha |\cdot ) & \propto p(Y,w|\alpha )p(\alpha |\tau )\\ \; & \propto \exp\left\{-\frac{1}{2}{\left(h^{\alpha }-X\alpha \right)}^{T}W\left(h^{\alpha }-X\alpha \right)\right\}\times \exp\left\{-\frac{1}{2}\alpha^{T}T^{-1}\alpha \right\}\\ \; & \propto \exp\left\{-\frac{1}{2}{\left(\alpha -\mu_{\alpha }\right)}^{T}{\left(\Sigma_{\alpha }\right)}^{-1}\left(\alpha -\mu_{\alpha }\right)\right\}, \end{array}$$

where $\Sigma_{\alpha }={\left(X^{T}WX+T^{-1}\right)}^{-1}$ and $\mu_{\alpha }=\Sigma_{\alpha }X^{T}Wh^{\alpha }$. Recognizing this as the kernel of a normal density, we find that

$$\alpha |\cdot ∼N(\mu_{\alpha },\Sigma_{\alpha }).$$

Next, we derive the full conditional of the local shrinkage parameters $\tau_{p}$, for p=1,…,P−1:

$$\begin{array}{cc} p(\tau_{p}|\cdot ) & \propto p\left(\alpha_{p}|\tau_{p}\right)p\left(\tau_{p}|\delta_{p}\right)\\ \; & \propto \exp\left\{-\frac{\tau_{p}^{-1}}{2}\alpha_{p}^{2}\right\}\times \exp\left\{-\tau_{p}\frac{\delta_{p}^{2}}{2}\right\}\\ \; & \propto \exp\left\{-\frac{\delta_{p}^{2}{\left(\tau_{p}^{-1}-\mu_{p}\right)}^{2}}{2{\left(\mu_{p}\right)}^{2}\tau_{p}^{-1}}\right\}, \end{array}$$

where $\mu_{p}=\sqrt{\delta_{p}^{2}/\alpha_{p}^{2}}$. Recognizing this as the kernel of an inverse-Gaussian density, we find that

$$\tau_{p}^{-1}|\cdot ∼Inv-Gaussian\left(\mu_{p},\delta_{p}^{2}\right),\;\mathrm{for}\;p=1,\ldots ,P-1.$$

Moreover, the full conditional distribution for the global shrinkage parameters $\delta_{p}$ is derived as follows. Exploiting the fact that the Laplace density is a scale mixture of normals with an exponential mixing density [22]:

$$\begin{array}{cc} p(\delta_{p}|\cdot ) & \propto p\left(\alpha_{p}|\tau_{p}\right)p\left(\tau_{p}|\delta_{p}\right)p\left(\delta_{p}\right)\\ \; & \propto \frac{\delta_{p}}{2}\exp\left\{-\delta_{p}\left|\alpha_{p}\right|\right\}\times {\left(\delta_{p}\right)}^{a_{\delta }-1}\exp\left\{-\delta_{p}b_{p}\right\}\\ \; & \propto {\left(\delta_{p}\right)}^{a_{\delta_{p}}^{*}-1}\exp\left\{-\delta_{p}\left(b_{\delta_{p}}^{*}\right)\right\}, \end{array}$$

where $a_{\delta_{p}}^{*}=a_{\delta }+1$ and $b_{\delta_{p}}^{*}=b_{\delta }+\left|\alpha_{p}\right|$. Recognizing this as the kernel of a Gamma density, we find that

$$\delta_{p}|\cdot ∼Gamma\left(a_{\delta_{p}}^{*},b_{\delta_{p}}^{*}\right),\;\mathrm{for}\;p=1,\ldots ,P-1.$$

We turn our attention to the random effects and derive the full conditional for the subject-specific random effects, $\gamma_{0}={\left(\gamma_{01},\ldots ,\gamma_{0N}\right)}^{T}$, where *N* is the number of participants in this study. Let $h^{0}={\left(h_{1}^{0},\ldots ,h_{N}^{0}\right)}^{T}$ where $h_{i}^{0}:=h_{i}-M_{i}\eta -X_{i}\alpha -1_{n_{i}}\gamma_{1k(i)}$, and let $Z_{0}=\mathrm{diag}\left(1_{n_{1}},\ldots ,1_{n_{N}}\right)$, where $n_{i}$ is the number of observations from the *i*th individual, i=1,…,N.

$$\begin{array}{cc} p(\gamma_{0}|\cdot ) & \propto p\left(Y,w|\nu \right)p\left(\gamma_{0}|\sigma_{0}^{2}\right)\\ \; & \propto \exp\left\{-\frac{1}{2}{\left(h^{0}-Z_{0}\gamma_{0}\right)}^{T}W\left(h^{0}-Z_{0}\gamma_{0}\right)\right\}\times \exp\left\{-\frac{1}{2}\gamma_{0}^{T}\left(\sigma_{0}^{-2}I_{n}\right)\gamma_{0}\right\}\\ \; & \propto \exp\left\{-\frac{1}{2}{\left(\gamma_{0}-\mu_{0}\right)}^{T}{\left(\Sigma_{0}\right)}^{-1}\left(\gamma_{0}-\mu_{0}\right)\right\}, \end{array}$$

where $\Sigma_{0}={\left(Z_{0}^{T}WZ_{0}+\sigma_{0}^{-2}I_{n}\right)}^{-1}$ and $\mu_{0}=\Sigma_{0}Z_{0}^{T}Wh^{0}$. Recognizing this as the kernel of a normal density, we find that

$$\gamma_{0}|\cdot ∼N\left(\mu_{0},\Sigma_{0}\right).$$

The full conditional distribution for the variance component of the subject-specific random effects, $\sigma_{0}^{2}$, is derived as follows.

$$\begin{array}{cc} p(\sigma_{0}^{2}|\cdot ) & \propto p\left(\gamma_{0}|\sigma_{0}^{2}\right)p\left(\sigma_{0}^{2}\right)\\ \; & {\left(\sigma_{0}^{-2}\right)}^{n/2}\exp\left\{-\frac{\sigma_{0}^{-2}}{2}\gamma_{0}^{T}\gamma_{0}\right\}\times {\left(\sigma_{0}^{-2}\right)}^{a_{0}-1}\exp\left\{-\sigma_{0}^{-2}b_{0}\right\}\\ \; & \propto {\left(\sigma_{0}^{-2}\right)}^{a_{0}^{*}-1}\exp\left\{-\sigma_{0}^{-2}\left(b_{0}^{*}\right)\right\}, \end{array}$$

where $a_{0}^{*}=a_{0}+N/2$ and $b_{0}^{*}=b_{0}+0.5\cdot \gamma_{0}^{T}\gamma_{0}$. Recognizing this as the kernel of a Gamma density, we find that

$$\sigma_{0}^{2}∼Inv-Gamma\left(a_{0}^{*},b_{0}^{*}\right).$$

Next, we derive the full conditional for the trial-specific random effects $\gamma_{1}={\left(\gamma_{11},\ldots ,\gamma_{1K}\right)}^{T}$, where *K* is the number of trials in this study. Let $h^{1}=h-M\eta -X\alpha -Z_{0}\gamma_{0}$, and let $Z_{1}=\mathrm{diag}{\left(1_{m_{1}},\ldots ,1_{m_{K}}\right)}^{T}$ where $m_{k}$ is the number of observations coming from trial *k*, k=1,…,K. Then,

$$\begin{array}{cc} p(\gamma_{1}|\cdot ) & \propto p\left(Y,w|\nu \right)p\left(\gamma_{1}|\sigma_{1}^{2}\right)\\ \; & \propto \exp\left\{-\frac{1}{2}{\left(h^{1}-Z_{1}\gamma_{1}\right)}^{T}W\left(h^{1}-Z_{1}\gamma_{1}\right)\right\}\times \exp\left\{-\frac{1}{2}\gamma_{1}^{T}\left(\sigma_{1}^{-2}I_{K}\right)\gamma_{1}\right\}\\ \; & \propto \exp\left\{-\frac{1}{2}{\left(\gamma_{1}-\mu_{1}\right)}^{T}{\left(\Sigma_{1}\right)}^{-1}\left(\gamma_{1}-\mu_{1}\right)\right\}, \end{array}$$

where $\Sigma_{1}={\left(Z_{1}^{T}WZ_{1}+\sigma_{1}^{-2}I_{K}\right)}^{-1}$ and $\mu_{1}=\Sigma_{1}Z_{1}^{T}Wh^{1}$. Recognizing this as the kernel of a normal density, we find that

$$\gamma_{1}|\cdot ∼N\left(\mu_{1},\Sigma_{1}\right).$$

The full conditional distribution for the variance component of the trial-specific random effects, $\sigma_{1}^{2}$, is derived as follows.

$$\begin{array}{cc} p(\sigma_{1}^{2}|\cdot ) & \propto p\left(\gamma_{1}|\sigma_{1}^{2}\right)p\left(\sigma_{1}^{2}\right)\\ \; & {\left(\sigma_{1}^{-2}\right)}^{K/2}\exp\left\{-\frac{\sigma_{1}^{-2}}{2}\gamma_{1}^{T}\gamma_{1}\right\}\times {\left(\sigma_{1}^{-2}\right)}^{a_{1}-1}\exp\left\{-\sigma_{1}^{-2}b_{1}\right\}\\ \; & \propto {\left(\sigma_{1}^{-2}\right)}^{a_{1}^{*}-1}\exp\left\{-\sigma_{1}^{-2}\left(b_{1}^{*}\right)\right\}, \end{array}$$

where $a_{1}^{*}=a_{1}+K/2$ and $b_{1}^{*}=b_{1}+0.5\cdot \gamma_{1}^{T}\gamma_{1}$. Recognizing this as the kernel of a Gamma density, we find that

$$\sigma_{1}^{2}∼Inv-Gamma\left(a_{1}^{*},b_{1}^{*}\right).$$

These full conditionals were used to construct an MCMC algorithm in the usual manner. To validate the proposed model and MCMC algorithm, an in-depth numerical study was conducted. This study was designed to emulate the primary features of the opioid data under study. The results (not shown) of this numerical study suggest that our proposed approach performs well and is appropriate for analyzing the motivating data.

## Appendix B. Efficacy and Safety Clinical Trials of Buprenorphine Maintenance Treatment

**Table A1:** Studies with Individual Patient Data Analyzed.

Division (Study ID)	Title	Investigators	Release Date
DTMC (CSP-999)	A Multicenter Clinical Trial of Buprenorphine in Treatment of Opiate Dependence	Walter Ling, M.D., Donald R. Wesson, M.D., C. James Klett, Ph.D.	2 September 2015
DTMC (CSP-1008A)	A Multicenter Efficacy/Safety Trial of Buprenorphine/Naloxone for the Treatment of Opiate Dependence	Peter Bridge, M.D., Paul J. Fudala, Ph.D.	4 December 2014
DTMC (CSP-1008B)	A Multicenter Safety Trial of Buprenorphine/Naloxone for the Treatment of Opiate Dependence	Peter Bridge, M.D.	4 December 2014
DTMC (CSP-1018)	A Multicenter Safety Trial of Buprenorphine/Naloxone for the Treatment of Opiate Dependence	Walter Ling, M.D., Paul J. Fudala, Ph.D., Paul Casadonte, M.D.	2 September 2015
CTN (CTN-0027)	Starting Treatment with Agonist Replacement Therapies (START)	Walter Ling, M.D.	30 July 2009
CTN (CTN-0030)	A Two-Phase Randomized Controlled Clinical Trial of Buprenorphine/Naloxone Treatment Plus Individual Drug Counseling for Opioid Analgesic Dependence	Walter Ling, M.D., Roger Weiss, M.D.	22 June 2011

## Appendix C. Model Analysis for CSP-999 Trial

### Appendix C.1. Patient Characteristics

The data consist of 15,983 urinalysis results from 654 subjects who participated in the CSP-999 trial. The number of urinalyses per subject ranged from 1 to 59, while the mean number of urinalyses per subject was 24.44 and the median was 17. Summaries of the demographic, sociodemographic, and substance use variables are given in Table A5. Several of the variables summarized in Table 1 are no longer available when only examining CSP-999 trial In particular, CSP-999 trial patients are either white, Hispanic, black, American Indian, or Asian and hence, the race categorized as “Other” was removed. None of the patients work for the military, so the work type category “Military” was removed. All of the patients have a stable living arrangement, so the “No Stable” living arrangement category was removed. All CSP-999 trial patients use heroine, so the “Heroine Use” categorical variable was removed. Finally, none of the patients’ chosen mode of opioid abuse was sublingual, so the “Sublingual” category for the mode of opiate abuse variable was removed. For ease of comparison, the reference group is the same as the one used for the full analysis. The mean age, income, and years of opioid use are 36.16 years, $19,454 per year and 11.56 years, respectively (presented in Table A5), while the mean dose is 7.49 mg/day (presented in Table A4).

### Appendix C.2. Functional Generalized Linear Mixed Model

Through the model in (1), we relate the daily dose patterns leading up to the clinic visit with urinalysis, while controlling for the 17 demographic, sociodemographic, and substance use variables detailed in Table A5. Note that, because all of our data comes from the CSP-999 trial, the trial-specific random effects $\gamma_{1k(i)}$ are removed from the model.

Figure A1 summarizes the estimated coefficient function $\hat{\beta }\left(t\right)$ (solid black line), which represents the buprenorphine daily dose effect for the 15 days leading up to a clinic visit with urinalysis. For comparison purposes, the coefficient function estimated from the full (all trials) analysis is also plotted (solid red line). The 95% credible intervals estimated from the full and reduced analyses are also plotted (red and black dashed line, respectively). Table A6 reports the demographic and substance use variables that were found to be significant. Of the 49 variable fixed effects, 5 were deemed to be important by the model (i.e., their estimated credible intervals did not contain zero). Table A6 summarizes these significant factors by reporting the estimated posterior mean (point estimate of the effect), estimated standard deviation of the posterior (measure of uncertainty), and 95% equal-tailed credible interval for each parameter. The analogous results for the full set of demographic and substance use variables are provided in Table A2 and Table A3.

**Table A2:** Analysis results: Summary includes the posterior mean estimates (Est), the estimated posterior standard deviations (ESE), and the estimated 95% equal-tailed credible intervals (CI95).

	All Trials			CSP-999		
**Variable**	**Est**	**ESE**	**CI95**	**Est**	**ESE**	**CI95**
Intercept	**2.05**	**0.27**	**(1.54, 2.61)**	1.49	0.77	(−0.07, 2.96)
Age	**−0.02**	**0.01**	**(−0.03, −0.01)**	−0.02	0.02	(−0.05, 0.03)
**Gender** (Ref: Male)						
Female	0.13	0.08	(−0.03, 0.30)	0.43	0.26	(−0.03, 0.94)
**Race** (Ref: White)						
Black	−0.16	0.14	(−0.43, 0.12)	**−1.27**	**0.32**	**(−1.89, −0.64)**
American Indian	0.14	0.27	(−0.39, 0.68)	−0.62	0.88	(−2.83, 0.83)
Asian	−0.14	0.27	(−0.72, 0.35)	−1.31	1.44	(−4.83, 0.67)
Hispanic	−0.22	0.12	(−0.46, 0.02)	0.02	0.24	(−0.47, 0.52)
Other	−0.32	0.67	(−1.79, 0.93)			
**Education** (Ref: High School)						
Graduate School	−0.34	0.24	(−0.81, 0.11)	−1.27	0.86	(−2.98, 0.21)
Standard College	0.05	0.16	(−0.25, 0.37)	−0.11	0.34	(−0.79, 0.59)
Partial College	−0.18	0.10	(−0.39, 0.02)	−0.04	0.21	(−0.47, 0.37)
Partial High School	−0.27	0.15	(−0.58, 0.01)	−0.03	0.27	(−0.63, 0.44)
Junior High School	−0.20	0.21	(−0.63, 0.18)	−0.18	0.34	(−0.98, 0.44)
Less than 7th Grade	−0.35	0.47	(−1.39, 0.47)	−0.48	0.84	(−2.53, 0.88)
**Emp. History** (Ref: Skilled)						
Never Gainfully	0.07	0.14	(−0.20, 0.34)	0.02	0.32	(−0.62, 0.70)
Unskilled	−0.20	0.16	(−0.52, 0.09)	−0.22	0.36	(−0.97, 0.44)
Machine Operator	−0.01	0.13	(−0.26, 0.24)	−0.18	0.28	(−0.76, 0.34)
Clerical/Sales	0.11	0.12	(−0.14, 0.35)	0.07	0.28	(−0.51, 0.64)
Administrative	−0.04	0.15	(−0.35, 0.24)	0.04	0.32	(−0.55, 0.73)
Business Manager	−0.24	0.24	(−0.72, 0.22)	−0.93	0.93	(−3.03, 0.48)
Executive	−0.18	0.26	(−0.71, 0.29)	0.05	1.04	(−2.03, 2.47)
**Work Type** (Ref: Full time)						
Regular PT	−0.10	0.15	(−0.39, 0.18)	−0.42	0.34	(−1.13, 0.21)
Irregular PT	0.07	0.13	(−0.19, 0.35)	0.29	0.30	(−0.25, 0.91)
Student	0.06	0.26	(−0.44, 0.61)	0.17	0.72	(−1.38, 1.67)
Military	−0.28	1.22	(−3.25, 2.02)			
Retired	0.23	0.24	(−0.19, 0.71)	0.21	0.79	(−1.13, 2.08)
Unemployed	**0.33**	**0.14**	**(0.05, 0.59)**	0.35	0.34	(−0.27, 1.05)
Controlled	0.84	0.59	(−0.2, 2.11)	1.18	0.97	(−0.30, 3.30)
Income	0.00	0.00	(0.00, 0.00)	0.00	0.00	(0.00, 0.00)
**Marital Status** (Ref: Married)						
Remarried	−0.17	0.39	(−1.01, 0.59)	0.56	0.93	(−1.07, 2.67)
Widowed	0.19	0.25	(−0.25, 0.73)	0.30	0.52	(−0.61, 1.38)
Separated	0.19	0.17	(−0.10, 0.55)	0.15	0.33	(−0.46, 0.81)
Divorced	0.04	0.12	(−0.20, 0.28)	0.09	0.28	(−0.44, 0.66)
Never Married	0.19	0.12	(−0.05, 0.42)	0.35	0.26	(−0.08, 0.89)
**Living Arr** (Ref: Partner and Child)						
Partner Only	0.24	0.13	(−0.02, 0.49)	**0.79**	**0.30**	**(0.17, 1.38)**
Child Only	−0.03	0.16	(−0.36, 0.29)	−0.19	0.31	(−0.82, 0.42)
Parents	0.21	0.17	(−0.10, 0.53)	0.28	0.29	(−0.23, 0.87)
Family	0.05	0.16	(−0.27, 0.36)	−0.22	0.34	(−0.96, 0.38)
Friends	−0.14	0.16	(−0.44, 0.17)	−0.07	0.30	(−0.74, 0.50)
Alone	−0.03	0.18	(−0.42, 0.31)	0.10	0.70	(−1.40, 1.60)
Controlled	−0.01	0.37	(−0.77, 0.75)	0.98	1.02	(−0.63, 3.24)
No Stable	−0.15	0.36	(−0.93, 0.52)			

**Table A3:** Analysis results: Summary includes the posterior mean estimates (Est), the estimated posterior standard deviations (ESE), and the estimated 95% equal-tailed credible intervals (CI95).

	All Trials			CSP−999		
**Variable**	**Est**	**ESE**	**CI95**	**Est**	**ESE**	**CI95**
**Heroine Use** (Ref: YES)						
NO	**−0.57**	**0.21**	**(−1.00, −0.16)**			
Years of Opiate Use	0.01	0.01	(−0.01, 0.02)	0.01	0.02	(−0.02, 0.04)
**Mode of Opioid Abuse** (Ref: IV)						
Oral	**−1.32**	**0.23**	**(−1.78, −0.88)**	**−1.21**	**0.48**	**(−2.11, −0.21)**
Snorting	−0.14	0.10	(−0.35, 0.06)	−0.15	0.23	(−0.60, 0.29)
Smoking	−0.40	0.28	(−0.94, 0.09)	−0.57	0.78	(−2.37, 0.69)
Sublingual	−1.34	0.97	(−3.42, 0.16)			
Other	0.02	0.41	(−0.82, 0.89)	−0.42	0.72	(−2.08, 0.74)
**Cocaine Use** (Ref: YES)						
NO	−0.04	0.09	(−0.22, 0.12)	−0.17	0.28	(−0.69, 0.37)
**Meth Use** (Ref: NO)						
YES	0.13	0.09	(−0.05, 0.31)	**0.77**	**0.28**	**(0.24, 1.31)**
**Alcohol Use** (Ref: YES)						
NO	−0.12	0.10	(−0.33, 0.07)	**−0.49**	**0.23**	**(−0.97, −0.04)**
**Tranquilizer Use** (Ref: NO)						
YES	0.07	0.10	(−0.14, 0.25)	0.38	0.24	(−0.06, 0.85)
**Marijuana Use** (Ref: YES)						
NO	0.05	0.10	(−0.16, 0.25)	0.20	0.23	(−0.27, 0.64)
**PCP Use** (Ref: NO)						
YES	−0.01	0.11	(−0.22, 0.21)	−0.07	0.28	(−0.60, 0.54)

**Table A4:** Treatment and outcome characteristics of individuals used in the trial CSP-999 analysis.

	Mean	Median	Range
Daily Dose	7.49	4	0–64
Time in Trial	148.76	133	3–527
Urinalysis (Yes = 1)	0.51	1	0–1

**Table A5:** Sociodemographic characteristics and drug use history for the individuals used in the CSP-999 trial analysis.

*Demographics *			*Sociodemographics*		
**Age**	Mean	SD	**Income**	Mean	SD
	36.16	7.78		19,454	21,365
**Gender**	N	%	**Employment History**	N	%
Male	453	69	Skilled Manual	151	23
Female	201	31	Never Gainfully	168	26
**Race**	N	%	Machine Operator	83	13
White	309	47	Clerical/Sales	124	19
Hispanic	184	28	Administrative	54	8
Black	152	23	Unskilled	64	10
American Indian	5	1	Business Manager	7	1
Asian	4	1	Executive	3	<1
Other	0	0	**Work Type**	N	%
			Full time	294	45
*Drug Use History*			Unemployed	198	30
**Years of Opiate Abuse**	Mean	SD	Irregular PT	81	12
	11.56	8.70	Regular PT	58	9
**Heroin Use**	N	%	Retired	7	1
YES	654	100	Student	7	1
NO	0	0	Controlled	9	1
**Mode of Opiate Abuse**	N	%	Military	0	0
IV	406	62	**Education**	N	%
Snort	194	30	High School	213	33
Oral	37	6	Partial College	201	31
Smoking	9	1	Partial High School	119	18
Other	8	1	Standard College	48	7
Sublingual	0	0	Junior High School	56	8
**Cocaine Use**	N	%	Complete Graduate School	11	2
YES	540	83	Less than 7th Grade	6	1
NO	114	17	**Marital Status**	N	%
**Meth Use**	N	%	Married	170	26
NO	436	67	Never Married	256	39
YES	218	33	Divorced	142	22
**Alcohol Use**	N	%	Separated	64	10
YES	446	68	Widowed	18	3
NO	208	32	Remarried	4	<1
**Tranquilizer Use**	N	%	**Living Arr**	N	%
NO	353	54	Partner and Child	163	25
YES	301	46	Partner Only	126	19
**Marijuana Use**	N	%	Parents	116	18
YES	482	74	Family	55	8
NO	172	26	Friends	97	15
**PCP Use**	N	%	Alone	8	1
NO	533	82	Child Only	82	13
YES	121	18	Controlled	7	1
			No Stable	0	0

**Table A6:** Sensitivity analysis results for CSP-999: Summary includes the posterior mean estimates (Est), the estimated posterior standard deviations (ESE), and the estimated 95% equal-tailed credible intervals (CI95) for the significant fixed effects.

Variable	Est	ESE	CI95
**Race** (Ref: White)			
Black	−11.27	0.32	(−11.89, −10.64)
**Living Arr** (Ref: Partner and Child)			
Partner Only	0.79	0.30	(0.17, 1.38)
**Mode of Opioid Abuse** (Ref: IV)			
Oral	−11.21	0.48	(−12.11, −10.21)
**Meth Use** (Ref: NO)			
YES	0.77	0.28	(0.24, 1.31)
**Alcohol Use** (Ref: YES)			
NO	−10.49	0.23	(−10.97, −10.04)

## References

[1] Kuehn B.M. (2021). Massive Costs of the US Opioid Epidemic in Lives and Dollars. JAMA.

[2] Kampman K., Jarvis M. (2015). American Society of Addiction Medicine (ASAM) National Practice Guideline for the Use of Medications in the Treatment of Addiction Involving Opioid Use. J. Addict. Med..

[3] Ling W., Jacobs P., Hillhouse M., Hasson A., Thomas C., Freese T., Sparenborg S., McCarty D., Weiss R., Saxon A. (2010). From research to the real world: Buprenorphine in the decade of the Clinical Trials Network. J. Subst. Abuse Treat..

[4] Mattick R.P., Breen C., Kimber J., Davoli M. (2014). Buprenorphine maintenance versus placebo or methadone maintenance for opioid dependence. Cochrane Database Syst. Rev..

[5] Fiellin D.A., Pantalon M.V., Chawarski M.C., Moore B.A., Sullivan L.E., O’Connor P.G., Schottenfeld R.S. (2006). Counseling plus buprenorphine-naloxone maintenance therapy for opioid dependence. N. Engl. J. Med..

[6] Bergen A.W., Baurley J.W., Ervin C.M., McMahan C.S., Bible J., Stafford R.S., Mudumbai S.C., Saxon A.J. (2022). Effects of buprenorphine dose and therapeutic engagement on illicit opiate use in opioid use disorder treatment trials. Int. J. Environ. Res. Public Health.

[7] Ramsay J., Silverman B. (2005). Functional Data Analysis.

[8] Hastie T., Tibshirani R., Friedman J. (2009). The Elemnts of Statistical Learning: Data Mining, Inference, and Prediction.

[9] Armagan A., Dunson D., Lee J. (2013). Generalized double Pareto shrinkage. Stat. Sin..

[10] Browne W.J., Draper D. (2006). A comparison of Bayesian and likelihood-based methods for fitting multilevel models. Bayesian Anal..

[11] Gelman A., Carlin J.B., Stern H.S., Dunson D.B., Vehtari A., Rubin D.B. (2013). Bayesian Data Analysis.

[12] Polson N., Scott J., Windle J. (2012). Bayesian inference for logistic models using Pólya-Gamma latent variables. J. Am. Stat. Assoc..

[13] Audigier V., Husson F., Josse J. (2016). A principal component method to impute missing values for mixed data. Adv. Data Anal. Classif..

[14] Crist R.C., Vickers-Smith R., Kember R.L., Rentsch C.T., Xu H., Edelman E.J., Hartwell E.E., Kampman K.M., Kranzler H.R. (2021). Analysis of genetic and clinical factors associated with buprenorphine response. Drug Alcohol Depend..

[15] Hser Y.I., Evans E., Grella C., Ling W., Anglin D. (2015). Long-term course of opioid addiction. Harv. Rev. Psychiatry.

[16] Weiss R.D., Potter J.S., Provost S.E., Huang Z., Jacobs P., Hasson A., Lindblad R., Connery H.S., Prather K., Ling W. (2010). A multi-site, two-phase, Prescription Opioid Addiction Treatment Study (POATS): Rationale, design, and methodology. Contemp. Clin. Trials.

[17] Dreifuss J.A., Griffin M.L., Frost K., Fitzmaurice G.M., Potter J.S., Fiellin D.A., Selzer J., Hatch-Maillette M., Sonne S.C., Weiss R.D. (2013). Patient characteristics associated with buprenorphine/naloxone treatment outcome for prescription opioid dependence: Results from a multisite study. Drug Alcohol Depend..

[18] Stefanski L.A. (2000). Measurement Error Models. J. Am. Stat. Assoc..

[19] Center for Disease Control and Prevention (2021). Drug Overdose Deaths in the U.S. Top 100,000 Annually. https://www.cdc.gov/nchs/pressroom/nchs_press_releases/2021/20211117.htm.

[20] Lapham G., Boudreau D.M., Johnson E.A., Bobb J.F., Matthews A.G., McCormack J., Liu D., Samet J.H., Saxon A.J., Campbell C.I. (2020). Prevalence and treatment of opioid use disorders among primary care patients in six health systems. Drug Alcohol Depend..

[21] Ma J., Bao Y.P., Wang R.J., Su M.F., Liu M.X., Li J.Q., Degenhardt L., Farrell M., Blow F.C., Ilgen M. (2019). Effects of medication-assisted treatment on mortality among opioids users: A systematic review and meta-analysis. Mol. Psychiatry.

[22] Park T., Casella G. (2008). The Bayesian Lasso. J. Am. Stat. Assoc..

